# 2,6-Diamino­pyridinium 2-carb­oxy­benzoate

**DOI:** 10.1107/S1600536810032903

**Published:** 2010-08-28

**Authors:** Mohammad T. M. Al-Dajani, Hassan H. Abdallah, Nornisah Mohamed, Mohd Mustaqim Rosli, Hoong-Kun Fun

**Affiliations:** a School of Pharmaceutical Sciences, Universiti Sains Malaysia, 11800 USM, Penang, Malaysia; b School of Chemical Sciences, Universiti Sains Malaysia, 11800 USM, Penang, Malaysia; c X-ray Crystallography Unit, School of Physics, Universiti Sains Malaysia, 11800 USM, Penang, Malaysia

## Abstract

In the crystal of the title mol­ecular salt, C_5_H_8_N_3_^+^·C_8_H_5_O_4_^−^, the diamino­pyridine cation and the phthalate anion are linked by a pair of N—H⋯O hydrogen bonds. Within the phthalate anion, an almost symmetrical O—H⋯O hydrogen bond is observed. The ion pairs are linked by further N—H⋯O hydrogen bonds, generating a two-dimensional network lying parallel to (10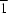).

## Related literature

For background to 2,6-diamino­pyridines, see: Abu Zuhri & Cox (1989 ▶); Inuzuka & Fujimoto (1990 ▶); El-Mossalamy (2001 ▶). For background and the biological activity of phthalic acid, see: Brike *et al.* (2002 ▶); Yamamoto *et al.* (1990 ▶). For related structures: see: Büyükgüngör & Odabąsoğlu (2006 ▶); Al-Dajani *et al.* (2009 ▶); Raissi Shabari *et al.* (2010 ▶). For the stability of the temperature controller used in the data collection, see: Cosier & Glazer (1986 ▶).
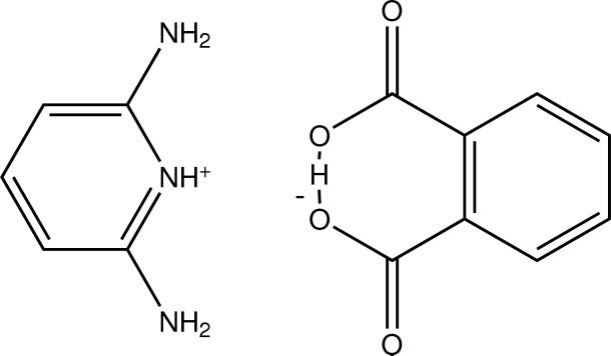


## Experimental

### 

#### Crystal data

C_5_H_8_N_3_^+^·C_8_H_5_O_4_^−^
*M* *_r_* = 275.26
Monoclinic, 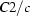

*a* = 32.332 (11) Å
*b* = 3.7246 (14) Å
*c* = 24.184 (8) Å
β = 123.036 (6)°
*V* = 2441.5 (15) Å^3^
*Z* = 8
Mo *K*α radiation
μ = 0.11 mm^−1^
*T* = 100 K
0.47 × 0.10 × 0.03 mm

#### Data collection

Bruker APEXII DUO CCD area-detector diffractometer
Absorption correction: multi-scan (*SADABS*; Bruker, 2009 ▶) *T*_min_ = 0.948, *T*_max_ = 0.996
5492 measured reflections
2757 independent reflections
1869 reflections with *I* > 2σ(*I*)
*R*_int_ = 0.041

#### Refinement

*R*[*F* ^2^ > 2σ(*F* ^2^)] = 0.053
*wR*(*F* ^2^) = 0.197
*S* = 1.06
2757 reflections
181 parameters
H-atom parameters constrained
Δρ_max_ = 0.41 e Å^−3^
Δρ_min_ = −0.40 e Å^−3^

### 

Data collection: *APEX2* (Bruker, 2009 ▶); cell refinement: *SAINT* (Bruker, 2009 ▶); data reduction: *SAINT*; program(s) used to solve structure: *SHELXTL* (Sheldrick, 2008 ▶); program(s) used to refine structure: *SHELXTL*; molecular graphics: *SHELXTL*; software used to prepare material for publication: *SHELXTL* and *PLATON* (Spek, 2009 ▶).

## Supplementary Material

Crystal structure: contains datablocks global, I. DOI: 10.1107/S1600536810032903/hb5557sup1.cif

Structure factors: contains datablocks I. DOI: 10.1107/S1600536810032903/hb5557Isup2.hkl

Additional supplementary materials:  crystallographic information; 3D view; checkCIF report

## Figures and Tables

**Table 1:** Hydrogen-bond geometry (Å, °)

*D*—H⋯*A*	*D*—H	H⋯*A*	*D*⋯*A*	*D*—H⋯*A*
O2—H1*O*2⋯O3	1.18	1.29	2.437 (3)	162
N1—H1*A*⋯O3	0.86	1.98	2.828 (3)	167
N2—H2*A*⋯O4	0.86	1.98	2.834 (3)	177
N2—H2*B*⋯O4^i^	0.86	2.10	2.851 (3)	145
N3—H3*A*⋯O2^ii^	0.86	2.35	3.042 (3)	138
N3—H3*B*⋯O1^iii^	0.86	2.01	2.858 (3)	171

Symmetry codes: (i) 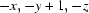; (ii) 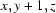; (iii) 
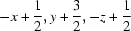
.

**Fig. 1.**
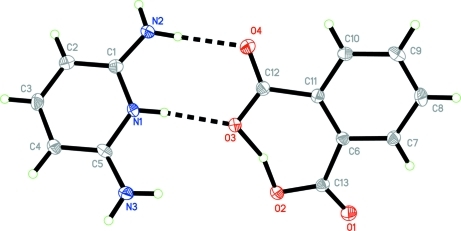
The molecular structure of (I), showing 50% probability displacement ellipsoids. Hydrogen atoms are shown as spheres of arbitrary radius. Dashed lines indicate the intermolecular hydrogen bonds.

**Fig. 2.**
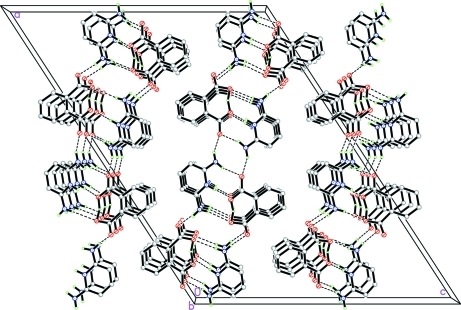
The crystal packing of (I) viewed down the b axis. Dashed lines indicate hydrogen bonds. H atoms not involved in the hydrogen bond interactions have been omitted for clarity.

## supplementary crystallographic information

### Comment

Phthalic acid is an aromatic dicarboxylic acid which can be used for the preparation of many organic and inorganic compounds such as dyes, perfumes and phthalates(Brike *et al.* 2002). Some of its derivatives have anti-tumor promoting action (Yamamoto *et al.* 1990). The diaminopyridine have an important role in the preparation of aromatic azo dyes (Abu Zuhri & Cox, 1989; Inuzuka & Fujimoto, 1990) and in many polarographic investigations (El-Mossalamy, 2001).

All geometrical parameters in the title compound, C_5_H_8_N_3_^+^.C_8_H_5_O_4_^-^,(I), are within normal ranges and are comparable with the related structures (Büyükgüngör & Odabąsoǧlu, 2006; Al-Dajani *et al.*, 2009; Shabari *et al.*, 2010). In the crystal structure, the anion and the cation were linked by N1—H1A···O3 and N2—H2A···O4 interactions. In the phthalate anion, a strong intramolecular interaction of O2—H1O2···O3 was observed. Intermolecular N—H···O hydrogen bonds (Table 1) further contribute to the stabilization of crystal structure, forming an infinite two-dimensional network parallel to the (101) plane.

### Experimental

In a round bottom flask, 25ml of THF was mixed with 2,6-diaminopyridine (0.01 mol, 1.1 g) with stirring.  Phthalic anhydrate (0.01 mol, 1.5 g) was dissolved in THF and then added in small portions into the round bottom flask and refluxed for 2 hours. The gray precipitate formed was filtrated and washed with THF. Brown plates of (I) were formed by dissolving the precipitate in hot water and letting it to cool at room temperature. The crystals was then filtered and dried at 60°C.

### Refinement

O-bound H atoms were located from a difference Fourier map and refined as riding with U_iso_(H) = 1.5U_eq_(O).The remaining H atoms were positioned geometrically [N–H = 0.86Å, C–H = 0.93 Å and refined using a riding model, with U_iso_(H) = 1.2U_eq_(N,C)].

### Crystal data

**Table:**

C_5_H_8_N_3_^+^·C_8_H_5_O_4_^−^	*F*(000) = 1152
*M**_r_* = 275.26	*D*_x_ = 1.498 Mg m^−^^3^
Monoclinic, *C*2/*c*	Mo *K*α radiation, λ = 0.71073 Å
Hall symbol: -C 2yc	Cell parameters from 975 reflections
*a* = 32.332 (11) Å	θ = 3.8–28.1°
*b* = 3.7246 (14) Å	µ = 0.11 mm^−^^1^
*c* = 24.184 (8) Å	*T* = 100 K
β = 123.036 (6)°	Plate, brown
*V* = 2441.5 (15) Å^3^	0.47 × 0.10 × 0.03 mm
*Z* = 8	

### Data collection

**Table:**

Bruker APEXII DUO CCD area-detector diffractometer	2757 independent reflections
Radiation source: fine-focus sealed tube	1869 reflections with *I* > 2σ(*I*)
graphite	*R*_int_ = 0.041
φ and ω scans	θ_max_ = 27.5°, θ_min_ = 3.4°
Absorption correction: multi-scan (*SADABS*; Bruker, 2009)	*h* = −36→42
*T*_min_ = 0.948, *T*_max_ = 0.996	*k* = −4→4
5492 measured reflections	*l* = −31→27

### Refinement

**Table:**

Refinement on *F*^2^	Primary atom site location: structure-invariant direct methods
Least-squares matrix: full	Secondary atom site location: difference Fourier map
*R*[*F*^2^ > 2σ(*F*^2^)] = 0.053	Hydrogen site location: inferred from neighbouring sites
*wR*(*F*^2^) = 0.197	H-atom parameters constrained
*S* = 1.06	*w* = 1/[σ^2^(*F*_o_^2^) + (0.1138*P*)^2^] where *P* = (*F*_o_^2^ + 2*F*_c_^2^)/3
2757 reflections	(Δ/σ)_max_ < 0.001
181 parameters	Δρ_max_ = 0.41 e Å^−^^3^
0 restraints	Δρ_min_ = −0.40 e Å^−^^3^

### Special details

**Table:**

Experimental. The crystal was placed in the cold stream of an Oxford Cyrosystems Cobra open-flow nitrogen cryostat (Cosier & Glazer, 1986) operating at 100.0 (1) K.
Geometry. All esds (except the esd in the dihedral angle between two l.s. planes) are estimated using the full covariance matrix. The cell esds are taken into account individually in the estimation of esds in distances, angles and torsion angles; correlations between esds in cell parameters are only used when they are defined by crystal symmetry. An approximate (isotropic) treatment of cell esds is used for estimating esds involving l.s. planes.
Refinement. Refinement of F^2^ against ALL reflections. The weighted R-factor wR and goodness of fit S are based on F^2^, conventional R-factors R are based on F, with F set to zero for negative F^2^. The threshold expression of F^2^ > 2sigma(F^2^) is used only for calculating R-factors(gt) etc. and is not relevant to the choice of reflections for refinement. R-factors based on F^2^ are statistically about twice as large as those based on F, and R- factors based on ALL data will be even larger.

### Fractional atomic coordinates and isotropic or equivalent isotropic displacement parameters (Å^2^)

**Table:**

	*x*	*y*	*z*	*U*_iso_*/*U*_eq_	
N1	0.09892 (7)	0.9810 (6)	0.16036 (9)	0.0153 (5)	
H1A	0.1109	0.8974	0.1388	0.018*	
N2	0.02334 (7)	0.8089 (7)	0.07262 (10)	0.0195 (5)	
H2A	0.0381	0.7329	0.0542	0.023*	
H2B	−0.0082	0.7882	0.0526	0.023*	
N3	0.17839 (7)	1.1355 (7)	0.24299 (10)	0.0210 (5)	
H3A	0.1883	1.0487	0.2192	0.025*	
H3B	0.1993	1.2268	0.2810	0.025*	
C1	0.04920 (8)	0.9585 (7)	0.13210 (11)	0.0158 (5)	
C2	0.02953 (9)	1.0896 (8)	0.16702 (11)	0.0179 (6)	
H2C	−0.0042	1.0754	0.1494	0.021*	
C3	0.06092 (9)	1.2402 (8)	0.22794 (12)	0.0191 (6)	
H3C	0.0478	1.3309	0.2510	0.023*	
C4	0.11140 (9)	1.2624 (8)	0.25640 (11)	0.0169 (5)	
H4A	0.1319	1.3636	0.2980	0.020*	
C5	0.13083 (8)	1.1287 (7)	0.22106 (11)	0.0153 (5)	
O1	0.26121 (6)	−0.0595 (6)	0.12827 (8)	0.0228 (5)	
O2	0.21888 (6)	0.2628 (6)	0.15685 (7)	0.0191 (4)	
H1O2	0.1816	0.4181	0.1225	0.029*	
O3	0.14264 (6)	0.6094 (5)	0.10279 (8)	0.0193 (5)	
O4	0.06986 (6)	0.5317 (6)	0.01065 (9)	0.0272 (5)	
C6	0.18695 (8)	0.1875 (7)	0.03925 (11)	0.0148 (5)	
C7	0.20221 (9)	0.0642 (8)	−0.00144 (12)	0.0170 (5)	
H7A	0.2336	−0.0336	0.0179	0.020*	
C8	0.17220 (9)	0.0825 (8)	−0.06960 (12)	0.0193 (6)	
H8A	0.1836	0.0045	−0.0955	0.023*	
C9	0.12537 (9)	0.2173 (8)	−0.09827 (11)	0.0195 (6)	
H9A	0.1045	0.2259	−0.1440	0.023*	
C10	0.10903 (9)	0.3408 (7)	−0.05943 (11)	0.0159 (5)	
H10A	0.0772	0.4316	−0.0797	0.019*	
C11	0.13903 (8)	0.3330 (7)	0.00959 (11)	0.0145 (5)	
C12	0.11507 (8)	0.4977 (7)	0.04302 (11)	0.0161 (5)	
C13	0.22537 (8)	0.1262 (7)	0.11219 (11)	0.0154 (5)	

### Atomic displacement parameters (Å^2^)

**Table:**

	*U*^11^	*U*^22^	*U*^33^	*U*^12^	*U*^13^	*U*^23^
N1	0.0160 (10)	0.0203 (12)	0.0105 (9)	0.0010 (9)	0.0079 (8)	−0.0016 (9)
N2	0.0140 (9)	0.0278 (13)	0.0146 (9)	−0.0007 (10)	0.0066 (8)	−0.0046 (10)
N3	0.0140 (9)	0.0340 (15)	0.0135 (9)	−0.0008 (10)	0.0064 (8)	−0.0042 (10)
C1	0.0162 (11)	0.0156 (13)	0.0147 (10)	0.0007 (10)	0.0079 (9)	0.0025 (10)
C2	0.0162 (11)	0.0185 (14)	0.0181 (11)	0.0005 (11)	0.0089 (10)	0.0018 (11)
C3	0.0236 (12)	0.0207 (14)	0.0175 (11)	0.0051 (12)	0.0141 (10)	0.0020 (11)
C4	0.0196 (11)	0.0175 (13)	0.0136 (11)	−0.0001 (11)	0.0091 (10)	−0.0004 (11)
C5	0.0170 (11)	0.0148 (13)	0.0111 (10)	−0.0031 (10)	0.0057 (9)	−0.0006 (10)
O1	0.0190 (9)	0.0286 (11)	0.0184 (8)	0.0056 (9)	0.0085 (7)	0.0023 (9)
O2	0.0170 (8)	0.0274 (11)	0.0127 (8)	0.0010 (8)	0.0078 (7)	−0.0007 (8)
O3	0.0155 (8)	0.0272 (11)	0.0151 (8)	0.0011 (8)	0.0084 (7)	−0.0045 (8)
O4	0.0152 (9)	0.0409 (13)	0.0235 (9)	−0.0007 (9)	0.0092 (7)	−0.0105 (10)
C6	0.0147 (10)	0.0162 (13)	0.0134 (10)	−0.0004 (10)	0.0076 (9)	0.0005 (10)
C7	0.0161 (11)	0.0185 (13)	0.0184 (11)	−0.0018 (11)	0.0107 (9)	−0.0010 (11)
C8	0.0261 (13)	0.0192 (14)	0.0191 (11)	−0.0028 (12)	0.0166 (10)	−0.0008 (11)
C9	0.0209 (12)	0.0205 (14)	0.0126 (10)	−0.0037 (11)	0.0062 (9)	−0.0003 (11)
C10	0.0141 (10)	0.0156 (13)	0.0162 (11)	−0.0024 (10)	0.0072 (9)	−0.0013 (10)
C11	0.0153 (10)	0.0127 (12)	0.0162 (11)	−0.0016 (10)	0.0090 (9)	−0.0017 (10)
C12	0.0159 (11)	0.0176 (14)	0.0161 (11)	−0.0023 (11)	0.0095 (9)	−0.0015 (11)
C13	0.0141 (10)	0.0166 (13)	0.0161 (11)	−0.0004 (10)	0.0086 (9)	0.0020 (10)

### Geometric parameters (Å, °)

**Table:**

N1—C1	1.364 (3)	O2—C13	1.310 (3)
N1—C5	1.368 (3)	O2—H1O2	1.1764
N1—H1A	0.8600	O3—C12	1.285 (3)
N2—C1	1.329 (3)	O3—H1O2	1.2919
N2—H2A	0.8600	O4—C12	1.232 (3)
N2—H2B	0.8600	C6—C7	1.397 (3)
N3—C5	1.326 (3)	C6—C11	1.413 (3)
N3—H3A	0.8600	C6—C13	1.522 (3)
N3—H3B	0.8600	C7—C8	1.385 (3)
C1—C2	1.393 (3)	C7—H7A	0.9300
C2—C3	1.373 (3)	C8—C9	1.372 (3)
C2—H2C	0.9300	C8—H8A	0.9300
C3—C4	1.386 (3)	C9—C10	1.383 (3)
C3—H3C	0.9300	C9—H9A	0.9300
C4—C5	1.399 (3)	C10—C11	1.401 (3)
C4—H4A	0.9300	C10—H10A	0.9300
O1—C13	1.217 (3)	C11—C12	1.521 (3)
			
C1—N1—C5	123.8 (2)	C12—O3—H1O2	100.1
C1—N1—H1A	118.1	C7—C6—C11	118.6 (2)
C5—N1—H1A	118.1	C7—C6—C13	112.8 (2)
C1—N2—H2A	120.0	C11—C6—C13	128.5 (2)
C1—N2—H2B	120.0	C8—C7—C6	122.3 (2)
H2A—N2—H2B	120.0	C8—C7—H7A	118.8
C5—N3—H3A	120.0	C6—C7—H7A	118.8
C5—N3—H3B	120.0	C9—C8—C7	118.9 (2)
H3A—N3—H3B	120.0	C9—C8—H8A	120.5
N2—C1—N1	116.2 (2)	C7—C8—H8A	120.5
N2—C1—C2	125.2 (2)	C8—C9—C10	120.2 (2)
N1—C1—C2	118.5 (2)	C8—C9—H9A	119.9
C3—C2—C1	118.6 (2)	C10—C9—H9A	119.9
C3—C2—H2C	120.7	C9—C10—C11	122.0 (2)
C1—C2—H2C	120.7	C9—C10—H10A	119.0
C2—C3—C4	122.6 (2)	C11—C10—H10A	119.0
C2—C3—H3C	118.7	C10—C11—C6	117.9 (2)
C4—C3—H3C	118.7	C10—C11—C12	113.7 (2)
C3—C4—C5	118.3 (2)	C6—C11—C12	128.3 (2)
C3—C4—H4A	120.8	O4—C12—O3	122.4 (2)
C5—C4—H4A	120.8	O4—C12—C11	118.4 (2)
N3—C5—N1	118.0 (2)	O3—C12—C11	119.1 (2)
N3—C5—C4	123.9 (2)	O1—C13—O2	120.6 (2)
N1—C5—C4	118.1 (2)	O1—C13—C6	119.3 (2)
C13—O2—H1O2	99.9	O2—C13—C6	120.0 (2)
			
C5—N1—C1—N2	179.6 (2)	C9—C10—C11—C6	−1.2 (4)
C5—N1—C1—C2	−0.7 (4)	C9—C10—C11—C12	177.1 (2)
N2—C1—C2—C3	−179.5 (3)	C7—C6—C11—C10	0.9 (4)
N1—C1—C2—C3	0.9 (4)	C13—C6—C11—C10	−175.8 (2)
C1—C2—C3—C4	−0.9 (4)	C7—C6—C11—C12	−177.1 (3)
C2—C3—C4—C5	0.7 (4)	C13—C6—C11—C12	6.2 (4)
C1—N1—C5—N3	−179.8 (2)	C10—C11—C12—O4	19.0 (4)
C1—N1—C5—C4	0.5 (4)	C6—C11—C12—O4	−162.9 (3)
C3—C4—C5—N3	179.8 (3)	C10—C11—C12—O3	−158.2 (2)
C3—C4—C5—N1	−0.4 (4)	C6—C11—C12—O3	19.9 (4)
C11—C6—C7—C8	0.5 (4)	C7—C6—C13—O1	−9.9 (3)
C13—C6—C7—C8	177.8 (3)	C11—C6—C13—O1	167.0 (3)
C6—C7—C8—C9	−1.8 (4)	C7—C6—C13—O2	172.4 (2)
C7—C8—C9—C10	1.6 (4)	C11—C6—C13—O2	−10.8 (4)
C8—C9—C10—C11	−0.1 (4)		

### Hydrogen-bond geometry (Å, °)

**Table:**

*D*—H···*A*	*D*—H	H···*A*	*D*···*A*	*D*—H···*A*
O2—H1O2···O3	1.18	1.29	2.437 (3)	162
N1—H1A···O3	0.86	1.98	2.828 (3)	167
N2—H2A···O4	0.86	1.98	2.834 (3)	177
N2—H2B···O4^i^	0.86	2.10	2.851 (3)	145
N3—H3A···O2^ii^	0.86	2.35	3.042 (3)	138
N3—H3B···O1^iii^	0.86	2.01	2.858 (3)	171

Symmetry codes: (i) −*x*, −*y*+1, −*z*; (ii) *x*, *y*+1, *z*; (iii) −*x*+1/2, *y*+3/2, −*z*+1/2.

## References

[bb1] Abu Zuhri, A. Z. & Cox, J. A. (1989). *Mikrochim. Acta*, **11**, 277–283.

[bb2] Al-Dajani, M. T. M., Salhin, A., Mohamed, N., Loh, W.-S. & Fun, H.-K. (2009). *Acta Cryst.* E**65**, o2931–o2932. 10.1107/S1600536809044468 PMC2971263 21578508

[bb3] Brike, G., Hirsch, M. & Franz, V. (2002). US Patent No. 636 838 9B1.

[bb4] Bruker (2009). *APEX2*, *SAINT* and *SADABS* Bruker AXS Inc., Madison, Wisconsin, USA.

[bb5] Büyükgüngör, O. & Odabąsoğlu, M. (2006). *Acta Cryst.* E**62**, o3816–o3818.

[bb12] Cosier, J. & Glazer, A. M. (1986). *J. Appl. Cryst.* **19**, 105–107.

[bb6] El-Mossalamy, E. H. (2001). *Pigm. Resin Technol.* **30**, 164–168.

[bb7] Inuzuka, K. & Fujimoto, A. (1990). *Bull. Chem. Soc. Jpn*, **63**, 216–220.

[bb8] Raissi Shabari, A., Safaeimovahed, M. & Pourayoubi, M. (2010). *Acta Cryst.* E**66**, o551. 10.1107/S1600536810004150 PMC2983748 21580321

[bb9] Sheldrick, G. M. (2008). *Acta Cryst.* A**64**, 112–122. 10.1107/S0108767307043930 18156677

[bb10] Spek, A. L. (2009). *Acta Cryst.* D**65**, 148–155. 10.1107/S090744490804362X PMC2631630 19171970

[bb11] Yamamoto, S., Nakadate, T., Aizu, E. & Kato, R. (1990). *Carcinogenesis*, **11**, 749–754. 10.1093/carcin/11.5.749 2110512

